# Syndrome métabolique chez les patients hypertendus dans le service cardiologie du CHU Yalgado Ouedraogo de Ouagadougou, Burkina Faso

**DOI:** 10.11604/pamj.2014.19.290.4028

**Published:** 2014-11-17

**Authors:** Georges Rosario Christian Millogo, André Samandoulougou, Nobila Valentin Yaméogo, Aristide Relwendé Yaméogo, Koudougou Jonas Kologo, Jean Yves Toguyeni, Patrice Zabsonré

**Affiliations:** 1 Service de Cardiologie du CHU Yalgado Ouédraogo, Ouagadougou, Burkina Faso

**Keywords:** HTA, syndrome métabolique, rétinopathie hypertensive, HBP, metabolic syndrom, hypertensive retinopathy

## Abstract

**Introduction:**

Le syndrome métabolique constitue de nos jours un véritable problème de santé publique. Le syndrome métabolique est le moteur d'une double épidémie mondiale de diabète type II et de maladies cardiovasculaires. L'objectif de notre étude est de décrire les aspects épidémiologiques, cliniques, para cliniques et évolutifs chez les hypertendus dans le service de Cardiologie du CHU Yalgado Ouédraogo.

**Méthodes:**

Il s'agissait d'une étude rétrospective sur une période de deux ans dans le service de cardiologie chez les patients hypertendus ayant un syndrome métabolique.

**Résultats:**

La fréquence du syndrome métabolique était de 17,5% des patients hypertendus. Le sex ratio était de 1,2. L’âge moyen des patients étaient de 56,1 ±10,7 ans. Les patients connus hypertendus étaient de 92,1% avec une durée moyenne d’évolution de l'HTA qui était de 8,7 ± 5,9 ans. Le suivi était irrégulier dans 60% cas et une rupture du traitement dans 37,1% des cas. La dyslipidémie était notée dans 84,2% des cas et le diabète dans 60,5% des cas. La PAS moyenne était de 184,3 ± 47,3 mmHg et la PAD moyenne était de 110,7 ± 27,7 mmHg. L'HTA était sévère dans 63,2% des cas. La glycémie moyenne était de 8,3 ± 4,3 mmol/L, le LDL cholestérol moyen était de 3,5 ± 1,0 mmol/L et le taux des triglycérides moyen était de 1,6 ± 1,1 mmol/L. L'HVG électrique était notée chez 76,3% des patients et échographique dans 58,8% des cas. Les atteintes viscérales étaient neurologique dans 44,5%, rénale dans 55,3% et cardiaque dans 31,2% des cas. Le nombre moyen d'antihypertenseurs était de 3,0 ± 1,0 et 76,3% ont reçu au moins une trithérapie antihypertensive. Le taux de mortalité était de 5,3%.

**Conclusion:**

Le syndrome métabolique est une pathologie qui pose la problématique de la définition qui n'est pas consensuelle d'une part et d'autre part du contrôle de ses éléments constitutifs surtout l'HTA.

## Introduction

Le syndrome métabolique constitue de nos jours un véritable problème de santé publique. C'est un groupe de facteurs de risque défini par la fédération internationale de diabète (FID) comme étant la présence d'une obésité centrale associée à aux moins deux des facteurs suivants que sont un taux de triglycérides élevé, un taux de HDL cholestérol bas, une hypertension artérielle et un taux élevé de glycémie veineuse [1]. Le syndrome métabolique est le moteur d'une double épidémie mondiale de diabète type II et de maladies cardiovasculaires. Les personnes ayant un syndrome métabolique sont exposées à un risque plus élevé de subir un infarctus du myocarde ou un accident vasculaire cérébral (AVC) et à un risque deux fois plus élevé d'en mourir par rapport aux personnes non atteintes. Le risque lié au syndrome est supérieur au risque de chaque composant pris séparément, justifiant l'individualisation de cette entité [1, 2]. L'identification de ces personnes est très importante afin de leur proposer un traitement adéquat. Au Burkina Faso, nous ne disposons pas actuellement de données sur le syndrome métabolique d'où l'intérêt de notre étude sur les aspects épidémiologiques, cliniques, para cliniques et évolutifs chez les hypertendus dans le service de Cardiologie du CHU Yalgado Ouédraogo.

## Méthodes

Il s'est agi d'une étude descriptive rétrospective de deux ans (1^er^ janvier 2011 au 31 décembre 2012) dans le service de cardiologie du CHU Yalgado OUEDRAOGO. Nous avons disposé des registres d'entrées et de sorties du service de cardiologie et des dossiers cliniques des patients. Ont été inclus, les patients de plus de 15 ans, hypertendus chez qui le diagnostic de syndrome métabolique a été retenu sur la base d'un dossier médical exploitable. Les cas de pré éclampsie et d’éclampsie n'ont pas été retenus dans cette étude. Les données recueillies sur une fiche de collecte individuelle ont été saisies et analysées à l′aide du logiciel EPI INFO version 7. Pour les variables quantitatives, la moyenne était calculée avec l′écart-type. Le test t de Student était utilisé pour la comparaison des moyennes lorsque les variances étaient homogènes. Les tests statistiques ANOVA et Chi-deux ont été utilisés pour comparer les proportions des variables qualitatives. Les tests statistiques donnant des valeurs de p < 0,05 étaient considérés comme significatifs.

### Définitions opérationnelles

La définition du syndrome métabolique était celle de la FID [1]. L'hypertrophie ventriculaire gauche (HVG) électrique a été définie par l'indice de Sokolow-Lyon ou par l'indice de Cornell et l'HVG échographique a été définie comme une masse du ventricule gauche indexée = 115 g/m^2^ chez l'homme et = 95 g/m^2^ chez la femme [3]. L'insuffisance rénale a été définie à partir de la clairance de la créatinine calculée par la formule de Cockcroft et Gault [4].

## Résultats

Durant la période d′étude, 1213 patients ont été hospitalisés dans le service de Cardiologie du CHU YO. Nous avons enregistré 304 cas d'HTA soit 25,1% des patients. Le syndrome métabolique a été recherché chez 217 patients hypertendus soit 71,4% de l'ensemble des patients hypertendus et nous avons noté 38 cas de syndrome métabolique soit 17,5%.

**Figure 1:**
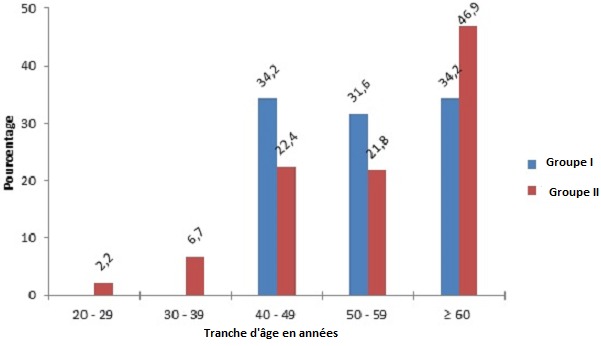
Répartition des patients en fonction de la tranche d’âge et du type d'HTA

Les patients hypertendus ont été répartis en deux groupes. Le Groupe I qui était constitué de patients ayant un syndrome métabolique, comptait 21 patients (55,3%) de sexe masculin, contre 17 (44,7%) de sexe féminin; soit un sex ratio de 1, 2. Le Groupe II qui correspondait aux autres patients hypertendus, était constitué de 111 (62%) patients de sexe masculin contre 68 (38%) de sexe féminin, soit un sex ratio de 1,6. Il n′y avait pas de différence statistiquement significative entre les sexes (p = 0,2). L’âge moyen de la population globale des hypertendus était de 57,6± 13,6 ans avec des extrêmes de 24 et 92 ans. L’âge moyen des patients du groupe I était de 56,1 ± 10,7 ans avec des extrêmes de 43 et 90 ans et celui du groupe II, de 57,9 ± 14,2 ans avec des extrêmes. La différence n’était pas statistiquement significative (p = 0,5). La Figure 1 montre la répartition des patients en fonction de la tranche d’âge et du type d'HTA. Les groupes socioprofessionnels les plus représentés étaient les salariés du public et/ou du privé dans le groupe I avec 11 cas (28,9%) suivis des commerçants avec 10 cas (26,3%). Dans le groupe II, les femmes au foyer étaient les plus nombreuses avec 53 cas (29,6%), suivies des salariés, 38 cas (21,2%). Les anciens patients étaient au nombre de 173 soit 79,7% de la population des hypertendus et étaient répartis comme suit: dans le groupe I, 35 (92,1%) patients étaient des anciens cas d'HTA contre trois (07,9%) nouveaux cas; et dans le groupe II, 138 (77,1%) patients étaient des anciens hypertendus contre 41 (22,9%) nouveaux cas d'HTA. La durée moyenne d’évolution de l'HTA de la population globale des hypertendus était de 7,9± 6,8 ans avec des extrêmes de 01 et 30 ans. La durée moyenne d’évolution de l'HTA des patients du groupe I était de 8,7 ± 5,9 ans avec des extrêmes de 01 et 24 ans et dans le groupe II, 7,6 ± 7,0 ans avec des extrêmes de 1 et 30 ans mais cette différence entre les deux groupes n’était pas statistiquement significative (p = 0,4). Parmi les anciens patients hypertendus du groupe I, le suivi était irrégulier dans 21 cas soit 60,0% des patients contre 90 cas, soit 65,2% pour les patients du groupe II mais la différence n’était pas significative (p = 0,3). Une rupture thérapeutique du traitement anti hypertenseur a été enregistrée dans 79 cas chez les anciens patients hypertendus, soit 45,7%.

Chez les anciens patients hypertendus du groupe I, 13 patients soit 37,1% avaient interrompu leur traitement contre 66 cas dans le groupe II. Une HTA familiale a été retrouvée chez 86 patients soit 39,6% de la population globale des patients hypertendus, dont 25 patients (65,8%) du groupe I. L'indice de masse corporel (IMC) moyen de la population globale des hypertendus était de 27,6 ± 18,3 kg/m^2^ avec des extrêmes de 14,7 et de 43,3. Dans le groupe I, nous avons noté un IMC moyen de 34,3 ± 3,5 kg/m^2^ avec des extrêmes de 30,1 et de 43,3. Dans le groupe II, il était de 26,3 ± 5,3 kg/m^2^ avec des extrêmes de 14,7 et de 43,1. Les patients du groupe I avaient un IMC moyen statistiquement supérieur à celui des patients du groupe II (p = 0,00). L'obésité était présente chez 71 patients soit 32,7% de la population générale, et une dyslipidémie a été retrouvée chez 32 patients tous du groupe I (84,2%). La PAS moyenne à l'admission dans la population globale des hypertendus était de 170,2 ± 44,1 mm Hg avec des extrêmes de 70 et de 300 et la PAD moyenne était de 104,6 ± 26,6 mm Hg avec des extrêmes de 50 et de 190 mm Hg. Chez les patients du groupe I, la PAS moyenne était de 184,3 ± 47,3 mm Hg avec des extrêmes de 100 et de 280 et la PAD moyenne était de 110,7± 27,7 mm Hg avec des extrêmes de 60 et de 170 mm Hg. Chez ceux du groupe II, la PAS moyenne était de 167,2± 43,0 mm Hg avec des extrêmes de 70 et de 300 et la PAD moyenne était de 103,4± 28,7 mm Hg avec des extrêmes de 50 et de 190 mm Hg. Les patients du groupe I avaient une PAS significativement plus élevée que celui des patients du groupe II (p = 0,03) tandis que la différence au niveau de la PAD n’était pas statistiquement significative entre les deux groupes. Sur le plan bilan biologique, une glycémie à jeun a été réalisée chez tous les patients, et le taux moyen de glycémie de la population globale d'HTA était de 6,3 ± 2,9 mmol/L avec des extrêmes de 2 et de 21 mmol/L. Dans le groupe I, nous avons noté un taux moyen de glycémie de 8,3 ± 4,3 mmol/L avec des extrêmes de 2 et de 21 mm Hg. Par contre, dans le groupe II, le taux moyen de glycémie de 5,9 ± 2,2 mmol/L avec des extrêmes de 2 et de 14. Les patients du groupe I avaient une glycémie significativement plus élevée que celui des patients du groupe II (p = 0,03).

Un diabète a été diagnostiqué chez 24 patients, soit 11,1% de la population globale des hypertendus, dont huit patients du groupe I. Le Tableau 1 montre la répartition des patients en fonction des facteurs de risque et du type d'HTA. Le bilan lipidique a aussi été réalisé chez tous les patients hypertendus, et le taux moyen de cholestérol total était de 4,7 ± 1,8 mmol/L avec des extrêmes de 1,1 et de 13. Dans le groupe I, nous avons noté un taux moyen de cholestérol total de 4,9 ± 1,4 mmol/L avec des extrêmes de 1,1 et de 7,8 mmol/L contre un taux moyen de 4,7 ± 1,8 mmol/L avec des extrêmes de 1,4 et de 13 mmol/L dans le groupe II mais cette différence n’était pas significative (p = 0,26). Le taux moyen de HDL cholestérol dans cette même population d'hypertendus était de 1,1 ± 0,4 mmol/L avec des extrêmes de 0,1 et de 3. Dans le groupe I, il était de 1,0 ± 0,4 mmol/L avec des extrêmes de 0,1 et de 1,91 mmol/L contre un taux de 1,1 ± 0,5 mmol/L avec des extrêmes de 0,3 et de 3 mmol/L dans le groupe II sans différence statistiquement significative entre les deux groupes (p = 0,8). Le taux moyen de LDL cholestérol chez les hypertendus de notre étude, était de 3,2 ± 1,6 mmol/L avec des extrêmes de 0,8 et de 11 mmol/L. Dans le groupe I, le taux moyen de LDL cholestérol était de 3,5 ± 1,0 mmol/L avec des extrêmes de 1,1 et de 4,7 mmol/L contre un taux moyen de 3,0 ± 1,7 mmol/L avec des extrêmes de 0,8 et de 11 mmol/L dans le groupe II. Les patients du groupe I avaient un taux de LDL cholestérol significativement plus élevée que celui des patients du groupe II (p = 0,02). Le taux moyen de triglycérides de la population globale d'HTA était de 1,3 ± 0,7 mmol/L avec des extrêmes de 0,4 et de 4,5 mmol/L. Il était de 1,6 ± 1,1 mmol/L avec des extrêmes de 0,4 et de 4,5 mmol/L chez les patients du groupe I contre 1,2 ± 0,5 mmol/L avec des extrêmes de 0,4 et de 3,0 mmol/L dans le groupe II. Les patients du groupe I avaient une triglycéridémie significativement plus élevée que celui des patients du groupe II (p = 0,02). Le diabète était plus fréquent chez les patients du groupe I ainsi que la dyslipidémie, comparés aux patients du groupe II (p = 0,00). Le taux moyen d'acide urique de la population globale des hypertendus était de 550,3 ± 241,6 µmol/L avec des extrêmes de 182 et de 1431 µmol/L.

**Tableau 1:** Répartition des patients en fonction des facteurs de risque et du type d'HTA

Facteurs de risque	Groupe I		Groupe II	
	Effectif	Pourcentage	Effectif	Pourcentage
Alcool	13	34,2	70	39,1
Tabac	06	15,2	32	17,9
Diabète	23	60,5	34	19,0
Dyslipidémie	32	84,2	84	46,9
Obésité	38	100,0	33	18,4

Dans le groupe I, nous avons noté un taux moyen d'acide urique de 509,9 ± 184,9 µmol/L avec des extrêmes de 182 et de 892 µmol/L et dans le groupe II, le taux moyen d'acide urique de 558,6± 251,5 µmol/L avec des extrêmes de 205 et de 1431 µmol/L. La différence n’était pas significative entre les deux groupes (p = 0,3). Nous avons noté 21 cas d'hyperuricémie soit 55,2% des patients du groupe I et 95 cas d'hyperuricémie soit 53,1% chez les patients du groupe II ayant bénéficié du dosage de l'acide urique. Tous nos patients ont bénéficié d'au moins un ECG au cours de leur hospitalisation. L'HVG était retrouvée dans 157 cas soit 72,4% des patients hypertendus. Le Tableau 2 montre la répartition des patients en fonction des différentes lésions de l'ECG et du type d'HTA. Le fond d’œil a été réalisé chez 125 patients hypertendus, dont 24 du groupe I. Il était normal dans 39 cas (31,2%), les atteintes oculaires les plus fréquentes étaient les rétinopathies hypertensives stades III avec 37 cas (29,6%) et les stades IV avec 23 cas (18,4%). Dans le groupe I, 87,5% (23 cas) des patients qui avaient bénéficié d'un fond d’œil étaient porteurs d'une atteint oculaire contre 64,4% des patients du groupe II. Le Tableau 3 montre la répartition des patients en fonction du fond d’œil et du type d'HTA.

**Tableau 2:** Répartition des patients en fonction des différentes lésions de l'ECG et du type d'HTA

ECG	Groupe I		Groupe II	
	Effectif	Pourcentage	Effectif	Pourcentage
**Normal**	01	02,6	11	06,1
**Hypertrophies**				
HVG	29	76,3	128	71,5
HAG	11	28,9	65	36,3
HAD	01	02,6	07	03 ?ç
**Troubles du rythme**				
ESV	01	02,6	26	14,5
ACFA	01	02,6	19	10,6
Flutter auriculaire	--	--	03	01,7
TV	--	--	03	01,7
FV	--	--	01	00,6
ESA	--	--	--	--
**Troubles de la conduction**				
BAV degré I	01	02,6	07	03,9
BAV degré III	01	02,6	01	00,6
BBDC	--	--	04	02,2
BBDI	02	05,3	05	02,8
HBAG	01	02,6	11	06,1
HBPG	--	--	01	00,6
BBGC	02	05,3	08	04,5
**Trouble de la repolarisation**	03	07,9	19	10,6
Nécrose	02	05,3	07	03,9

**Tableau 3:** Répartition des patients en fonction du fond d’œil et du type d'HTA

Fond œil	Groupe I		Groupe II	
	Effectif	Pourcentage	Effectif	Pourcentage
Normal	03	12,5	36	35,6
Stade I	01	04,2	08	07,9
Stade II	03	12,5	14	13,9
Stade III	13	54,2	24	23,8
Stade IV	04	16,6	19	18,8
Total	24	100,0	101	100,0

L’échocardiographie Doppler a été réalisée chez 123 patients soit 56,7% de la population globale d'hypertendus dont 20 patients (52,6%) du groupe I. Le diamètre télédiastolique du ventricule gauche (DTDVG) moyen de la population globale d'HTA était de 52,5 ± 9,6 mm avec des extrêmes de 35 et 76. Le DTDVG moyen était de 49,0 ± 6,8 mm chez les patients du groupe I avec des extrêmes de 37 et 61 contre 53,2 ± 10,0 mm avec des extrêmes de 35 et 76 mm chez ceux du groupe II sans différence significative (p = 0,07). Les index de contractilités calculés donnaient les résultats suivants: la fraction d’éjection (FE) moyenne de la population globale d'HTA était de 58,8 ± 17,1% avec des extrêmes de 20 et de 89%. Dans le groupe I, nous avons noté une FE moyenne de 69,2 ± 11,5% avec des extrêmes de 40 et de 89% contre une FE moyenne de 56,7 ± 17,3% avec des extrêmes de 19 et de 89% dans le groupe II. La FE était significativement plus basse chez les patients du groupe II que chez les patients du groupe I (p = 0,002). La FE était normale dans 17 cas (85%) des patients du groupe I, et dans 48 cas (47,6%) des patients du groupe II. La fraction de raccourcissement (FR) moyenne de la population globale d'HTA était de 31,3 ± 11,3% avec des extrêmes de 9 et de 52%. Elle était de 38,5 ± 11,3% avec des extrêmes de 20 et de 52% dans le groupe I, contre 29,9 ± 11,2% avec des extrêmes de 9 et de 52% dans le groupe II. La FR était significativement plus basse chez les patients du groupe II que chez les patients du groupe I (p = 0,002). La FR était normale dans 18 cas (90,0%) des patients du groupe I et dans 49 cas (51,5%) des patients du groupe II. L’épaisseur télédiastolique du septum interventriculaire (ETD SIV) moyenne chez nos patients hypertendus était de 12,4 ± 3,4 mm avec des extrêmes de 6 et de 29 mm. Dans le groupe I, elle était de 14,2 ± 3,6 mm avec des extrêmes de 10 et de 25 mm et dans le groupe II de 12,1 ± 3,3 mm avec des extrêmes de 6 et de 29 mm. Les patients du groupe I avaient une ETD SIV significativement plus élevée chez les patients du groupe II (p = 0,01). L’épaisseur télédiastolique de la paroi postérieure du VG (ETD PP) moyenne était de 11,2 ± 2,6 mm avec des extrêmes de 5 et de 19 dans la population globale des hypertendus. Elle était de 11,8 ± 2,0 mm avec des extrêmes de 8 et de 15 mm dans le groupe I et de 11,1 ± 2,7 mm avec des extrêmes de 5 et de 19 mm dans le groupe II mais la différence n’était pas statistiquement significative (p = 0,9).

La masse ventriculaire gauche indexée (MVGi) moyenne de la population globale des patients hypertendus était de 141,3 ± 48,9 g/m^2^ avec des extrêmes de 42,7 et de 337,4 g/m^2^. Dans le groupe I, la MVGi moyenne était de 129,1 ± 44,2 g/m^2^ avec des extrêmes de 71,0 et de 257,9 g/m^2^ contre 143,6 ± 49,6 g/m^2^ avec des extrêmes de 42,7 et de 337,4 g/m^2^. Dans le groupe II mais la différence n’était pas statistiquement significative (p= 0,22).

L'HVG était présente dans 69 cas soit 56,1% des patients de la population globale d'HTA. Parmi les patients du groupe I, elle était présente chez 14 patients soit 70,0%. L'HVG concentrique représentait 42,9% des HVG dans ce même groupe. Les troubles de la contractilité myocardique, à type d'hypokinésie, avaient été retrouvés chez trois patients (15%) du groupe I, et chez 52 patients (50,5) du groupe II. L'HTA sévère était notée dans 24 cas soit 63,2% des patients du groupe I. Les atteintes viscérales ont été retrouvées chez 30 patients (78,9%) du groupe I et chez 151 patients (84,4%) du groupe II. Elles étaient essentiellement des atteintes neurologiques dans 17 cas (44,7%), cardiovasculaires dans 12 cas (31,6%) et rénales dans 21 cas (55,3%) dans le groupe I. La répartition des atteintes neurologiques dans les deux groupes est résumée dans le Tableau 4. Les patients du groupe I présentaient beaucoup plus des atteintes neurologiques que les patients du groupe II (p = 0,01). Par contre, les patients du groupe II présentaient plus d′atteinte cardiovasculaire que les patients du groupe I: 112 cas (62,6%) contre 12 cas (31,6%) (p = 0,0003). La créatininémie a été dosée chez 96,8% des patients (n = 210) et chez tous les patients du groupe I. La créatininémie moyenne de la population globale des patients hypertendus était de 183,4 ± 184,7 µmol/L avec des extrêmes de 35 et de 1172 µmol/L. Dans le groupe I, elle était de 157,5 ± 136,8 µmol/L (extrêmes 50 et 806 µmol/L) contre 189,1 ± 193,6 µmol/L (extrêmes 35 et 1172 µmol/L) dans le groupe II, la différence n’était pas statistiquement significative (p = 0,34). En revanche, le diagnostic d'IR a été posé chez 21 (55,3%) patients du groupe I contre 133 (77,3) patients du groupe II (p = 0,01). Le Tableau 5 illustre la répartition des patients en fonction du stade de l'IR et du type d'HTA. L'IR était modérée chez neuf patients soit 42,9% des patients du groupe I.

**Tableau 4:** Répartition des patients en fonction de l'atteinte neurologique et du type d'HTA

Atteinte neurologique	Groupe I		Groupe II	
	Effectif	Pourcentage	Effectif	Pourcentage
AVC hémorragique	05	29,4	14	28,6
AVC ischémique	10	58,8	32	65,3
Encéphalopathie hypertensive	02	11,8	03	06,1
Total	17	100,0	49	100,0

**Tableau 5:** Répartition des patients en fonction du stade de l'insuffisance rénale et du type d'HTA

Insuffisance rénale	Groupe I		Groupe II	
	Effectif	Pourcentage	Effectif	Pourcentage
IR débutante	09	42,9	49	36,8
IR modérée	08	38,0	49	36,8
IR sévère	03	14,3	21	15,8
IR terminale	01	04,8	14	10,5
Total	21	100,0	133	100,0

Sur le plan thérapeutique, l’évaluation de la prise en charge a concerné l'usage de drogues antihypertenseurs. Le nombre moyen de médicaments utilisés dans la population générale était de 2,7 ± 0,9 (extrêmes deux et cinq). Il était de 3,0 ± 1,0 (extrêmes deux et cinq) chez les patients du groupe I contre 2 ± 0,9 (extrêmes zéro et cinq). L'association de plusieurs médicaments antihypertenseurs était significativement plus importante chez les patients du groupe I (p = 0,01). Parmi les patients du groupe I, 18 patients soit 47,4% avaient reçu une trithérapie antihypertensive. Dans ce même groupe, les molécules les plus utilisées étaient des IEC, dans 33 cas (86,8%), suivis des diurétiques, dans 31 cas (81,6%) et des inhibiteurs calciques, dans 27 cas (71%), cette distribution était sensiblement la même dans le groupe II. Le nombre moyen de jours d'hospitalisation de nos patients était de 11,5 ± 7,0 jours avec des extrêmes de un et de 36 jours. Ce nombre moyen de jours d'hospitalisation était de 12,0 ± 6,5 jours avec des extrêmes de deux et 25 jours dans le groupe I, contre 11,4 ± 7,1 jours avec des extrêmes de un et 36 jours dans le groupe II. La différence n’était pas significative (p = 0,66).

L’évolution sous traitement était favorable dans 195 cas et il y avait 24 décès (11,1%). Ce taux de mortalité était de 5,3% (deux cas) dans le groupe I, contre 12,3% (22 cas) dans le groupe II; la différence n’était pas statistiquement significative (p = 0,16).

## Discussion

### Limites et contraintes de l′étude

La principale difficulté de cette étude est liée à la définition du syndrome métabolique. En effet, nous avons été confrontés à trois définitions: la définition de National Cholesterol Panel /Adult Treatment Panel III (NCP/ATP III), de l'Organisation Mondiale de la Santé (OMS) et de la FID. Le caractère rétrospectif de l’étude ne nous avait pas permis d’évaluer les aspects évolutifs du syndrome métabolique à long terme. En plus, notre étude comportait un biais de recrutement du fait que nos patients étaient hospitalisés alors qu'une partie importante des patients hypertendus sont suivis en ambulatoire. Le site de notre étude ne nous permettait pas de généraliser les résultats de notre travail à l′ensemble de la population.

### Fréquence

La prévalence du syndrome métabolique dans notre étude était de 17,5%. Foucan [5] notait une prévalence de 11,3% dans les Caraïbes, Barrios en Espagne [6] et Mulè en Italie [7] trouvaient respectivement une prévalence de 52% et 37%. Au Nigéria, une étude réalisée chez les patients hypertendus notait une prévalence de 31,5% [8]. Cette différence des résultats pourrait s'expliquer par la pluralité de la définition du syndrome métabolique, les caractéristiques socio-économiques différentes entre les pays où les études sont réalisées et le fait que les patients hypertendus sont suivis dans la plupart du temps en ambulatoire. Notre échantillon était constitué de patients hospitalisés pour des complications d'HTA dans la plupart des cas. L’équipe de Desprès [2] explique cette forte prévalence du syndrome métabolique par la transition épidémiologique dans ces pays en développements, caractérisée par le changement du mode de vie actuel, basé sur une réduction de l'exercice physique, tandis que les apports caloriques sont en augmentation. D'où une augmentation de la graisse abdominale à haut niveau calorique qui va induire de nombreux désordres métaboliques bien répertoriés dans ce syndrome.

### Sexe

Le sexe masculin représentait 55,3% de la population soit un sex ratio de 1,2. Foucan [5] et Osuji au Nigéria [8] notaient une prédominance féminine. Barrios [6] et Mulè [7] notaient respectivement une prévalence du sexe masculin dans 56,1% et 47,7% des cas. Les aspects polygénétiques, la situation géographique de la population et le mode de vie sont des facteurs pouvant entraîner une disparité de la répartition en fonction du sexe. Dans notre contexte, les femmes auraient une faible accessibilité aux soins de santé du fait de leur faible pouvoir d'achat.

### Age

L’âge moyen de notre population était de 56,1 ans et 78,5% avaient un âge supérieur ou égal à 50 ans. On notait une augmentation de la prévalence avec l’âge. Foucan [5], Barrios [6] et Mulè [7] trouvaient un âge moyen respectif de 38 ans, 62,3 ans et 48,5 ans. Il a été démontré que la prévalence du syndrome métabolique augmentait avec l’âge. Cela pourrait s'expliquer par le fait que tous les niveaux de la pathogénèse du syndrome métabolique sont affectés par l'avancée de l’âge. Ainsi, l’âge est associé à une évolution de l'insulinorésistance, de l'altération d'autres hormones et à l'augmentation du tissu adipeux abdominal [9].

### Pression artérielle

La PAS moyenne était de 184,3 mmHg et la PAD moyenne était de 110,7 mmHg. L'HTA était sévère dans 63,2% des cas. Barrios [6] et Mulè [7] notaient respectivement une PAS moyenne de 145,8 mmHg et 162,6 mmHg, une PAD moyenne de 86 mmHg et 94,6 mmHg. D'une manière générale, on notait un mauvais contrôle des chiffres tensionnels chez les patients ayant un syndrome métabolique. En effet Barrios dans son étude [6] faisait le même constat. Une étude japonaise [10] montrait une bonne corrélation entre la prévalence du syndrome métabolique et le niveau de pression artérielle. Cette étude avait classé les patients en trois sous-groupes et avait noté une prévalence du syndrome métabolique de 9,9% chez les patients normo-tendus, 19,2% chez les pré hypertendus et 35,5% chez les hypertendus [10]. L’élévation des chiffres tensionnels chez les patients ayant un syndrome métabolique serait due à l'activation d'une cascade d'anomalies touchant de nombreux facteurs de risque [2].

### Biologie

La glycémie, le LDL cholestérol et les triglycérides étaient statistiquement plus élevés chez les patients ayant un syndrome métabolique. Mulè [7] et Barrios [6] notaient des résultats similaires dans leurs études respectives. La présence de ces facteurs de risque expose les patients aux complications cardiovasculaires et à une évolution rapide vers le diabète de type II. En plus, ces facteurs de risque, quand ils sont associés, accélèrent l’évolution de l'HTA vers les atteintes viscérales [2].

### Atteintes viscérales

Une atteinte neurologique, cardiaque ou rénale a été notée dans 78,9% des cas dans notre étude. Les atteintes neurologiques étaient présentes dans 44,5% des cas dont un AVC ischémique dans 58,8% des cas. Ces atteintes viscérales étaient statistiquement plus fréquentes chez les patients ayant un syndrome métabolique. Les complications cardiovasculaires sont augmentées d'un facteur 2 à 3 chez les patients ayant un syndrome métabolique et le risque d’évolution vers le diabète qui va lui-même aggraver ce risque cardiovasculaire [2]. Des études montrent que le syndrome métabolique est un facteur de risque indépendant d’événement cardiovasculaire chez les patients hypertendus [11, 12]. Le syndrome métabolique pourrait être un outil important pour les cliniciens pour la détection des patients ayant un risque faible à modéré pour l’évaluation traditionnelle des facteurs de risque. Ces patients pourront bénéficier d'un traitement agressif avec un contrôle rigoureux de la tension artérielle et des désordres métaboliques [11, 12].

### Utilisation des médicaments anti hypertenseurs

La consommation des médicaments anti hypertenseurs était très importante chez les patients ayant un syndrome métabolique. La moyenne des médicaments anti hypertenseurs était de 3 médicaments et 94,8% des patients avaient une bithérapie. Barrios [6] notait également des résultats similaires aux nôtres. Ces résultats pourraient s'expliquer par le contrôle difficile des chiffres tensionnels chez les patients ayant un syndrome métabolique. Cette augmentation de la consommation de médicaments entraîne une augmentation des dépenses de santé pour des populations qui ont déjà des revenus faibles dans notre contexte.

## Conclusion

Le syndrome métabolique est une pathologie qui pose le problème d'une part de sa définition jusque-là non consensuelle et d'autre part celui du contrôle de ses éléments constitutifs, surtout l'HTA. Le syndrome métabolique prédit mieux le risque cardiovasculaire que l'analyse de chaque facteur de risque pris isolément. Faire le diagnostic de syndrome métabolique chez un patient s'apparente donc à calculer son risque cardiovasculaire global. Il augmente en effet le risque de survenue de l'atteinte viscérale chez les patients hypertendus. Le meilleur traitement reste la prévention surtout dans les pays comme le Burkina Faso.
